# Inhibition of *Spodoptera frugiperda* phenoloxidase activity by the products of the *Xenorhabdus* rhabduscin gene cluster

**DOI:** 10.1371/journal.pone.0212809

**Published:** 2019-02-22

**Authors:** Maria Eugenia Nuñez-Valdez, Anne Lanois, Sylvie Pagès, Bernard Duvic, Sophie Gaudriault

**Affiliations:** 1 Universidad Autónoma del Estado de Morelos, Morelos, Mexico; 2 DGIMI, INRA, Université de Montpellier, Montpellier, France; Institute of Plant Physiology and Ecology Shanghai Institutes for Biological Sciences, CHINA

## Abstract

We evaluated the impact of bacterial rhabduscin synthesis on bacterial virulence and phenoloxidase inhibition in a *Spodoptera* model. We first showed that the rhabduscin cluster of the entomopathogenic bacterium *Xenorhabdus nematophila* was not necessary for virulence in the larvae of *Spodoptera littoralis* and *Spodoptera frugiperda*. Bacteria with mutations affecting the rhabduscin synthesis cluster (Δ*isnAB* and Δ*GT* mutants) were as virulent as the wild-type strain. We then developed an assay for measuring phenoloxidase activity in *S*. *frugiperda* and assessed the ability of bacterial culture supernatants to inhibit the insect phenoloxidase. Our findings confirm that the *X*. *nematophila* rhabduscin cluster is required for the inhibition of *S*. *frugiperda* phenoloxidase activity. The *X*. *nematophila* Δ*isnAB* mutant was unable to inhibit phenoloxidase, whereas Δ*GT* mutants displayed intermediate levels of phenoloxidase inhibition relative to the wild-type strain. The culture supernatants of *Escherichia coli* and of two entomopathogenic bacteria, *Serratia entomophila* and *Xenorhabdus poinarii*, were unable to inhibit *S*. *frugiperda* phenoloxidase activity. Heterologous expression of the *X*. *nematophila* rhabduscin cluster in these three strains was sufficient to restore inhibition. Interestingly, we observed pseudogenization of the *X*. *poinarii* rhabduscin gene cluster via the insertion of a 120 bp element into the *isnA* promoter. The inhibition of phenoloxidase activity by *X*. *poinarii* culture supernatants was restored by expression of the *X*. *poinarii* rhabduscin cluster under the control of an inducible P_*tet*_ promoter, consistent with recent pseudogenization. This study paves the way for advances in our understanding of the virulence of several entomopathogenic bacteria in non-model insects, such as the new invasive *S*. *frugiperda* species in Africa.

## Introduction

Insects rely on innate immune responses to defend themselves against foreign microorganisms. Their cellular defense mechanisms are mediated by hemocytes, the immunity cells of insects. Hemocytes play a key role in the phagocytosis, nodulation and encapsulation of intruding pathogens. The main humoral mechanisms involve antimicrobial peptides and the prophenoloxidase (PO) system [1]. The PO system is responsible for melanization, a process in which an insoluble brown-black pigment, melanin, is synthesized and deposited. Melanization takes place in three steps. The first of these steps is the recognition of pathogen-associated molecular patterns (PAMPs), such as the peptidoglycans or lipopolysaccharides of bacteria and the β-1,3-glucans of fungi. In the second step, a precursor, prophenoloxidase, is cleaved by a serine protease cascade to generate the active enzyme, phenoloxidase. In the third step, phenoloxidase catalyzes the oxidation of phenolic compounds, which then polymerize to form melanin. Melanin seals the wound (hemolymph clotting) and traps the intruding microorganisms (nodulation and encapsulation) [2–5]. Moreover, the polymerization of melanin generates redox-active melanogenic intermediates. These intermediates, alone or together with reactive intermediates of oxygen and nitrogen, are highly cytotoxic [6].

The importance of phenoloxidase activity to insect defense is highlighted by the strategies developed by various insect pathogens to circumvent this phenomenon and, thus, the melanization response. Virulence factors inhibiting the conversion of prophenoloxidase into the active enzyme phenoloxidase have been described in parasitoid wasps. These factors include a serine protease ortholog synthesized by *Cotesia rubecula* [7] and the serine proteinase inhibitors produced by the polyDNA virus of *Microplitis demolitor* and *Leptopilina boulardi* [8–11]. Other pathogens generate aromatic compounds capable of interacting directly with activated phenoloxidase. For example, the fungal metabolite kojic acid, produced by *Aspergillus* and *Penicillium* species, and the fusaric and picolinic acids produced by *Fusarium* spp. are potent inhibitors of phenoloxidase [12]. The entomopathogenic bacteria *Photorhabdus* and *Xenorhabdus*, which can access to their insect prey through their symbiotic nematodes [13–15], also have a wealth of phenoloxidase inhibitors (see below).

*Photorhabdus* and *Xenorhabdus* can interact directly with the insect immune system following their transfer from the nematode gut to the insect hemolymph [16–18]. They target the hemocytes with hemolysins [19], block the activity of antimicrobial peptides [20], and inhibit prophenoloxidase activation and eicosanoid-mediated nodulation [21]. *Photorhabdus* and *Xenorhabdus* kill the insect rapidly, allowing their symbiotic host nematodes to grow and reproduce in the insect cadaver [13,14,22].

Several phenoloxidase activity inhibitors have been identified in *Photorhabdus* and *Xenorhabdus*: 4-hydroxystilben, benzylideneacetone, 1,2-benzenedicarboxylic acid, benzaldehyde and rhabduscin [23–27]. The most potent of these inhibitors, rhabduscin, is a tyrosine-derived amidoglycosyl- and vinyl-isonitrile product. It inhibits both mushroom tyrosinase and insect phenoloxidase from waxmoth larvae (*Galleria mellonella*) at low nanomolar concentrations [27]. It is currently thought that it inhibits phenoloxidase by mimicking the substrate, associating non-covalently at the surface of the bacterial cell surface [27]. Rhabduscin has also been implicated in the virulence of *Xenorhabdus nematophila* in *G*. *mellonella*, which is highly susceptible to this bacterium [27].

Rhabduscin biosynthesis is dependent on three genes: *isnA* and *isnB*, which encode proteins involved in isonitrile biosynthesis, and *GT*, which encodes glycosyltransferase [28]. In *Xenorhabdus nematophila* ATCC19061^T^, these three genes are located in a single cluster, the heterologous overexpression of which confers rhabduscin production by *E*. *coli*. By contrast, in *Photorhabdus luminescens* TT01, the *GT* gene is located elsewhere in the genome and has a tandem duplication [28]. Interestingly, homologs of key rhabduscin synthesis genes, *isnAB*, have been identified in many bacteria from diverse groups, including a number of bacteria pathogenic to vertebrates, such as *Vibrio cholera*, *Aeromonas* spp., *Burkholderia pseudomallei*, *Pseudomonas aeruginosa*, and *Serratia marcescens* [29]. Moreover, the aglycone precursor of the rhabduscin synthesized by the IsnA and IsnB products encoded by *Photorhadus asymbiotica*, which causes opportunistic infections in humans, acts as a potent inhibitor of the mammalian alternative complement pathway [29]. These findings suggest an important role for the *isnAB* genes in host-pathogen interactions.

We investigated the importance of rhabduscin for the process of insect infection further, by evaluating the impact of *Xenorhabdus nematophila* rhabduscin synthesis on virulence and phenoloxidase activity in insects of agronomic importance from the genus *Spodoptera*. This genus includes *Spodoptera frugiperda* (*Sf*), a new invasive species in Africa [30], for which we developed an assay of phenoloxidase activity. This assay was also used to evaluate the impact of *X*. *nematophila* rhabduscin synthesis on phenoloxidase activity following heterologous expression in several members of the Enterobacteriaceae (*Escherichia coli*, *Serratia entomophila* and *Xenorhabdus poinarii*). We then focused on *X*. *poinarii*, in which we observed pseudogenization of the endogenous *X*. *poinarii* rhabduscin genes through an insertion into the promoter region.

## Materials and methods

### Bacterial growth conditions

Bacteria were routinely grown in Luria–Bertani (LB) broth, on 1.5% nutrient agar (Difco) plates at 28°C for *Xenorhabdus nematophila* ATCC19061^T^ (*Xn*), *Xenorhabdus poinarii* G6 (*Xp*) and *Serratia entomophila* MOR4.1 (*Se*) and at 37°C for *Escherichia coli*. The bacteria were stored in 16% glycerol (v/v) at -80°C. When required, kanamycin was added to the culture medium: 20 μg mL^-1^ final concentration for *Ec* and *Se*; or 40 μg mL^-1^ final concentration for *Xp*. P*tet* promoters were induced by adding anhydrotetracycline (aTc) at a final concentration of 100 ng mL^-1^ two hours after culture initiation (OD_540nm_ = 0.1). The strains used in this study are listed in Table 1.

**Table 1:** Strains and plasmids used in this study.

	Description	Source or reference
**Strains**		
*Xn*	*Xenorhabdus nematophila* ATCC19061^T^ wild-type strain isolated from *Steinernema carpocapsae* All nematode collected in the USA	[31]
*Xn* Δ*isnAB*^1^	*X*. *nematophila* ATCC19061^T^ *isnA*-*isnB* (vinyl-isocyanide biosynthetic genes) deletion mutant	[27]
*Xn* Δ*GT*^1^	*X*. *nematophila* ATCC19061^T^ XNC1_1223 (glycosyltransferase–encoding gene) deletion mutant	[27]
*Xp*	*Xenorhabdus poinarii* G6 strain isolated from *Steinernema glaseri* SK29 nematodes collected in North Carolina, USA	[32]
*Se*	*Serratia entomophila* Mor4.1 wild-type strain isolated from a dead third-instar *Phyllophaga blanchardi* larva collected from a cornfield (Morelos, Mexico).	[33]
*Ec *	*Escherichia coli* XL1Blue strain Δ(*mcrA*)*183* Δ(*mcrCB-hsdSMR-mrr*)*173 endA1 supE44 thi-1 recA1* gyrA96 relA1 lac *[F’* proAB lacI^q^*Z*Δ*M15 Tn*10 *(Tet*^*r*^*)]*	Stratagene
*E*. *coli* WM3064	*Escherichia coli* WM3064 strain derived from B2155, which is auxotrophic for DAP and used for conjugation experiments; *thrB1004 pro thi rpsL hsdS lacZΔM15 RP4-1360 Δ(araBAD)567 ΔdapA1341*::*[erm pir]*	[34]
**Plasmids**		
pGJ907 (= P*tet*-MCS)	Cloning vector, P_*tet*_ promoter, Tet^R^, Km^R^	[35]
p[*isnAB*-*GT*-*Xn*]	*isn*AB-*GT* cluster (XNC1_1221-XNC1_1223) from *X*. *nematophila* ATCC19061^T^ inserted between the *Kpn*I and *Bam*HI sites of the pGJ907 cloning vector, under the control of the P*tet* promoter, Km^R^	This work
p[*isnAB*-*GT*-*Xp*]	*isn*AB-*GT* cluster (XPG1_0843-XPG1_0845) from *X*. *poinarii* G6 inserted between *Eco*RI blunt-ended and *Sal*I sites in the pGJ907 cloning vector, under the control of the P*tet* promoter, Km^R^	This work

^1^ The integrity of the mutants was checked by PCR.

### Insect rearing and pathology assays

*S*. *littoralis* (*Sl*) and *S*. *frugiperda* (*Sf*) larvae were reared in DGIMI insectarium (Montpellier, France) on an artificial diet [36] at 23±1°C, with a photoperiod of 16 hours light:8 hours darkness and a relative humidity of 40 ± 5%. Pathogenicity experiments were performed by injecting a suspension of bacteria in the exponential growth phase (10^3^ bacteria/20 μL of LB broth) into 20 fifth-instar larvae, as previously described [37]. Three independent pathogenicity assays were performed for each bacterial strain in the *S*. *littoralis* pathoassay, facilitating statistical comparisons of mortality in a Wilcoxon test implemented in SPSS V18.0 (SPSS, Inc., Chicago, IL), as previously described [38]. Due to difficulties relating to the lack of standardization of pathoassays for *S*. *frugiperda*, we were unable to perform statistical analyses for this species, for which one representative experiment is, therefore, shown. The number of bacterial cells injected was checked by plating on nutrient agar and direct counts of the colonies formed.

### Nucleic acid manipulations

Plasmid DNA was extracted from *E*. *coli* with the GenElute^TM^HP Plasmid miniprep purification kit, as recommended by the manufacturer (Sigma). Restriction enzymes and T4 DNA ligase were used as recommended by the manufacturer (New England Biolabs and Promega, respectively). Oligonucleotide primers were synthesized by Eurogentec (Seraing, Belgium). PCR was performed in a T100 thermal cycler (Biorad) with the iProof high-fidelity DNA polymerase (Biorad). Amplified DNA fragments were purified with a PCR purification kit (Roche) and separated by electrophoresis in 1% agarose gels after digestion. Hydrolyzed DNA fragments were extracted from agarose gels with the NucleoTrap kit from Macherey-Nagel. All constructs were confirmed by DNA sequencing (MWG).

### Plasmid and recombinant strain construction

For the construction of p[*isnAB*-*GT*-*Xn*], the *isnAB*-*GT* locus from *Xn* genome was amplified by PCR with the primers L-Kpn-isnA (5’-GCGGTACCGGTTAGTGATGTGGAGGCAATAC-3’) and R-Bam-GT (5’-GCGGATCCGGATCTTATGCCGATTGAGC-3’) and inserted into the pGJ907 vector hydrolyzed with *Eco*RI and *Bam*HI. For the construction of p[*isnAB*-*GT*-*Xp*], the *isnAB*-*GT* locus from the *Xp* genome was amplified by PCR with the primers L-isnA-G6 (5’-GTAACAAGCTGAAAGAGAACGGATA-3’) and R-GT-G6-SalI (5’-GGCGTCGACGAAGGCGTCCGTGAAAAA-3’) and inserted into the pGJ907 vector hydrolyzed with *Eco*RI (blunt-ended) and *Bam*HI. Plasmids pGJ907, p[*isnAB*-*GT*-*Xn*] and p[*isnAB*-*GT*-*Xp*] were introduced into *Se*, *E*. *coli* XL1-Blue (*Ec*) and *E*. *coli* WM3064 by transformation. The WM3064 transformants were used to transfer plasmids pGJ907, p[*isnAB*-*GT*-*Xn*] and p[*isnAB*-*GT*-*Xp*] to *Xp* by conjugative mating, as previously described [39].

### Sf phenoloxidase inhibition assay

*Sf* hemocyte lysates were prepared at 4°C. Hemolymph from 60 *Sf* larvae was collected in 1.5 mL ice-cold Eppendorf tubes containing anticoagulant (69 mM KCl, 27 mM NaCl, 2 mM NaHCO_3_, 100 mM D-glucose, 30 mM tripotassium citrate, 26 mM citric acid, 10 mM Na_2_–EDTA, pH 4.6, 420 mOsm) [40]. The hemocytes were centrifuged at 800 x *g* for 1 min at 4°C and the supernatant was removed. The cell pellet was washed briefly with PBS (GIBCO) and the hemocytes were frozen in liquid nitrogen. The hemocyte lysate was then resuspended in 500 μL of 10 mM sodium cacodylate, pH 7, homogenized with a glass piston homogenizer and centrifuged at 16,000 x *g* for 30 minutes at 4°C. The hemocyte lysate supernatant (HLS) was stored at -20°C and subsequently used for the evaluation of phenoloxidase activity. Protein concentration of HLS was determined with the Bradford method using bovine serum albumin as standard [41].

The *Sf* phenoloxidase inhibition assay was performed at room temperature, in a 96-well microplate. An aliquot of 20 μL of HLS containing about 20 μg of proteins was first incubated for 5 minutes with 20 μL of α-chymotrypsin (5 mg mL^-1^), to activate the prophenoloxidase, and 20 μL of LB (HLS+) or bacterial culture supernatant at room temperature. For the negative control (HLS-), 20 μL of 10 mM sodium cacodylate, pH 7, was used instead of α-chymotrypsin. We then added 500 μL of L-DOPA (4 mg mL^-1^). Phenoloxidase activity was monitored by measuring the change in absorbance at 490 nm as a function of time with a microplate reader (Infinite M200 TECAN). The differences among samples were assessed by the Tukey-Kramer Multiple Comparison Test. The null hypothesis (Ho) for this test states that there are no differences among the means of the compared samples. The null hypothesis was contrasted against the alternative hypothesis (Ha) indicating that there are differences among the means of the samples evaluated. The GraphPad InStat3 software was used to make the statistical tests.

For the preparation of bacterial culture supernatants, bacteria carrying pGJ907 or plasmids derived from it were grown overnight in 5 mL of LB supplemented with 40 μg mL^-1^ kanamycin, at 28°C, with shaking at 250 rpm. We used 250 μL of this overnight culture to inoculate 50 mL of LB supplemented with 40 μg mL^-1^ kanamycin, which was then incubated in similar conditions. Anhydrotetracycline (aTc) was added to a final concentration of 100 ng mL^-1^ when the culture reached an OD_540_ of 0.1. After 24 hours of culture, 2 mL aliquots were removed and centrifuged at 14,400 x *g* at room temperature, passed through a filter with 0.22 μm pores and stored at -80°C until use.

### RNA preparation

Total RNA was extracted from cells cultured in LB broth to an OD_540nm_ of 0.7 with the RNeasy miniprep Kit (Qiagen), according to the manufacturer’s instructions. An additional incubation with DNase I (Qiagen) was performed. The quantity and quality of RNA were assessed with an Agilent 2100 Bioanalyzer, with the RNA 6000 Nano LabChip kit. We checked for the absence of DNA by carrying out PCR on each RNA preparation.

### RT-qPCR analysis

Reverse transcription followed by quantitative PCR (RT-qPCR) was performed as previously described [35] on the RNA samples obtained as described above. Briefly, for cDNA synthesis, the SuperScript II reverse transcriptase (Invitrogen) was used on 0.5 μg of total RNA with random hexamers (100 ng μL^-1^; Roche Diagnostics). qPCR analyses were performed with the SYBR green Master kit (Roche Diagnostics), 1 μL of a 1:50 dilution of cDNA and specific gene primers (1 μM) targeting internal regions within *recA* from *Xp* (L-recA-G6: AAACATCCGTCTGTTGCTATCC / R- recA-G6: CTCTCCAGCAGGCAGTTATTG), within *isnA* from *Xp* (L-isnA-G6: ATACACGAAGAAGAAGGTGTTTCAG / R- isnA-G6: TACCTGTCTGTTCAGTTTCTCCAAC), within *recA* from *Xn* (L-recA-ATCC19061 : ATTAATACTCTGGGAGAGTTGATCG / R-recA-ATCC19061 : AGTTTCTTATTCAACTCAGCAGCAG) and within *isnA* from *Xn* (L-isn-ATCC19061 : TCCTCGTAAAGTATTGGGTAAGATG / R-isnA-ATCC19061 : GTCATCAACATGAATGAGATCACTG). The reactions were performed in triplicate, with heating at 95°C for 10 min, followed by 45 cycles of 95°C for 5 s, 61°C for 10 s, and 72°C for 15 s and monitoring with a LightCycler 480 system (Roche). Melting curves were analyzed, and each curve contained a single peak. The data analyzed with the LightCycler 480 software are presented as a ratio with respect to the reference housekeeping gene *recA*, as previously described [35].

### Genomic analysis

We used the Genoscope microscopy platform (http://www.genoscope.cns.fr/agc/microscope/home/) to identify the *isnAB*-*GT* rhabduscin gene clusters and their putative promoter regions in the *Xn*, *X*. *bovienii* CS03, *X*. *bovienii* SS-2004, *Xp*, *X*. *doucetiae* FRM16 genome sequences. We used MultAlin, with the default parameters, to align the putative promoter regions [42].

## Results

### The *X*. *nematophila* rhabduscin gene cluster and bacterial virulence in two *Spodoptera* species

**Fig 1:**
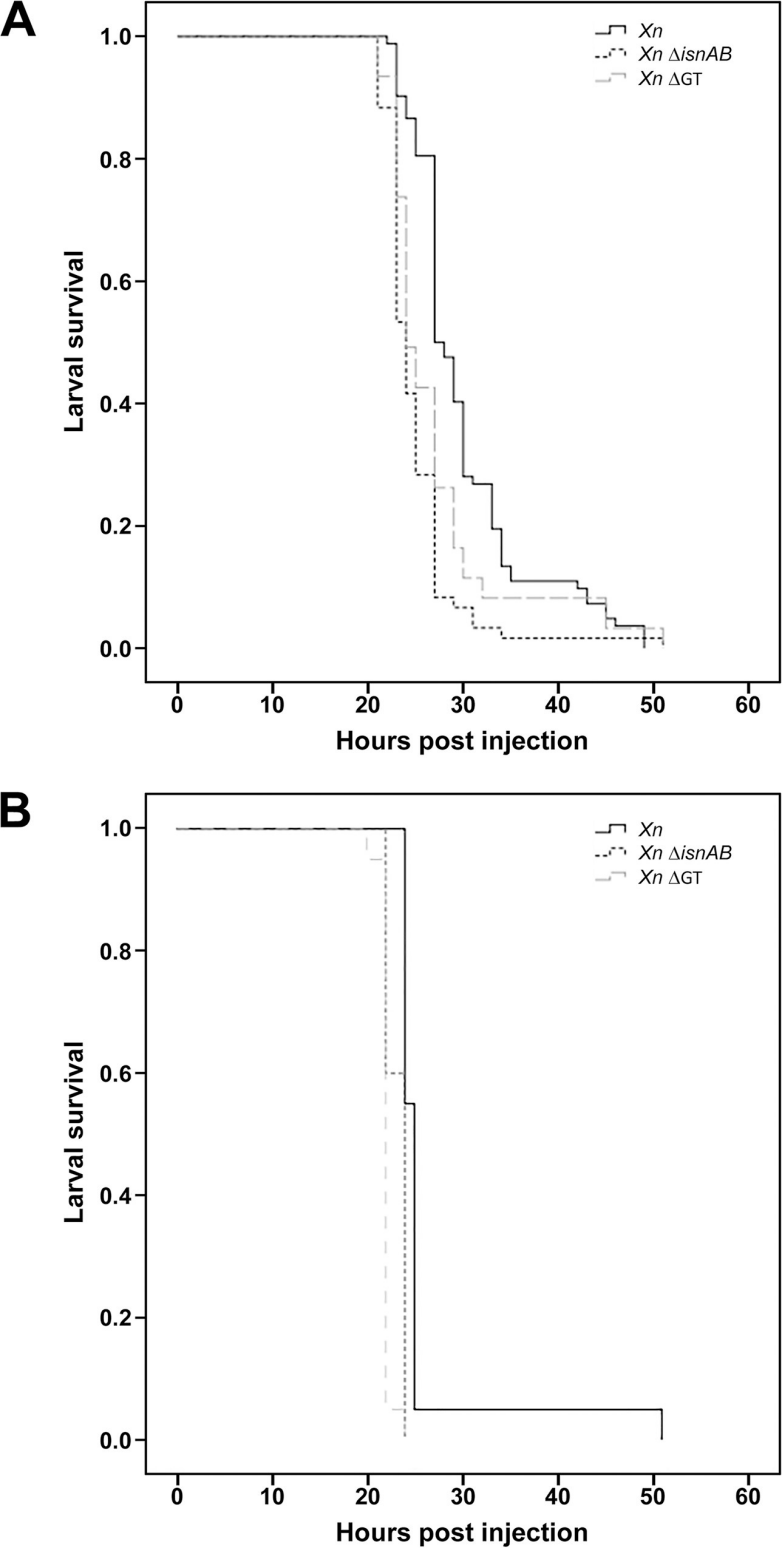
**Survival curves for *Spodoptera littoralis* (A) and *Spodoptera frugiperda* (B) after injection of the *X*. *nematophila* ATCC19061**^**T**^ **wild-type strain, or of the Δ*isnAB* or Δ*GT* mutant**. Pathogenicity assays were performed on *S*. *littoralis* and *S*. *frugiperda* larvae. As the *S*. *littoralis* assay had already been standardized, we show a cumulative curve of three independent experiments and we performed Wilcoxon tests to check for an absence of significant differences (*P*<0.05). As the *S*. *frugiperda* assay is not standardized, we were unable to perform statistical tests and we show a single representative curve.

It has been suggested that the *X*. *nematophila* rhabduscin gene cluster contributes to pathogenesis in the larvae of the highly susceptible species *G*. *mellonella* (waxmoth) [27]. However, this result was based on findings for a small number of individuals, with no statistical analysis. We were unable to standardize pathology assays on *G*. *mellonella* because it was difficult to determine the instar of the larvae. We therefore evaluated the effect of the rhabduscin cluster on bacterial virulence in two lepidopteran pests, the noctuid moth *Spodoptera littoralis* (*Sl*) and *Spodoptera frugiperda* (*Sf*). The pathology assay for *Spodoptera littoralis* (*Sl*) was standardized some time ago in our laboratory (see for example [16,37,43]). We injected *X*. *nematophila* ATCC19061^T^ wild-type strain (*Xn*), the *X*. *nematophila* ATCC19061^T^ Δ*GT* mutant (*Xn* Δ*GT*) and the *X*. *nematophila* ATCC19061^T^ Δ*isnAB* mutant (*Xn* Δ*isnAB*) into fifth-instar larvae of these two insects. Both mutants had a virulence similar to or slightly lower than that of the wild-type strain *Xn* in both species (Fig 1A and 1B).

### The *X*. *nematophila* rhabduscin gene cluster and the inhibition of *Sf* phenoloxidase by *X*. *nematophila* supernatants

**Fig 2:**
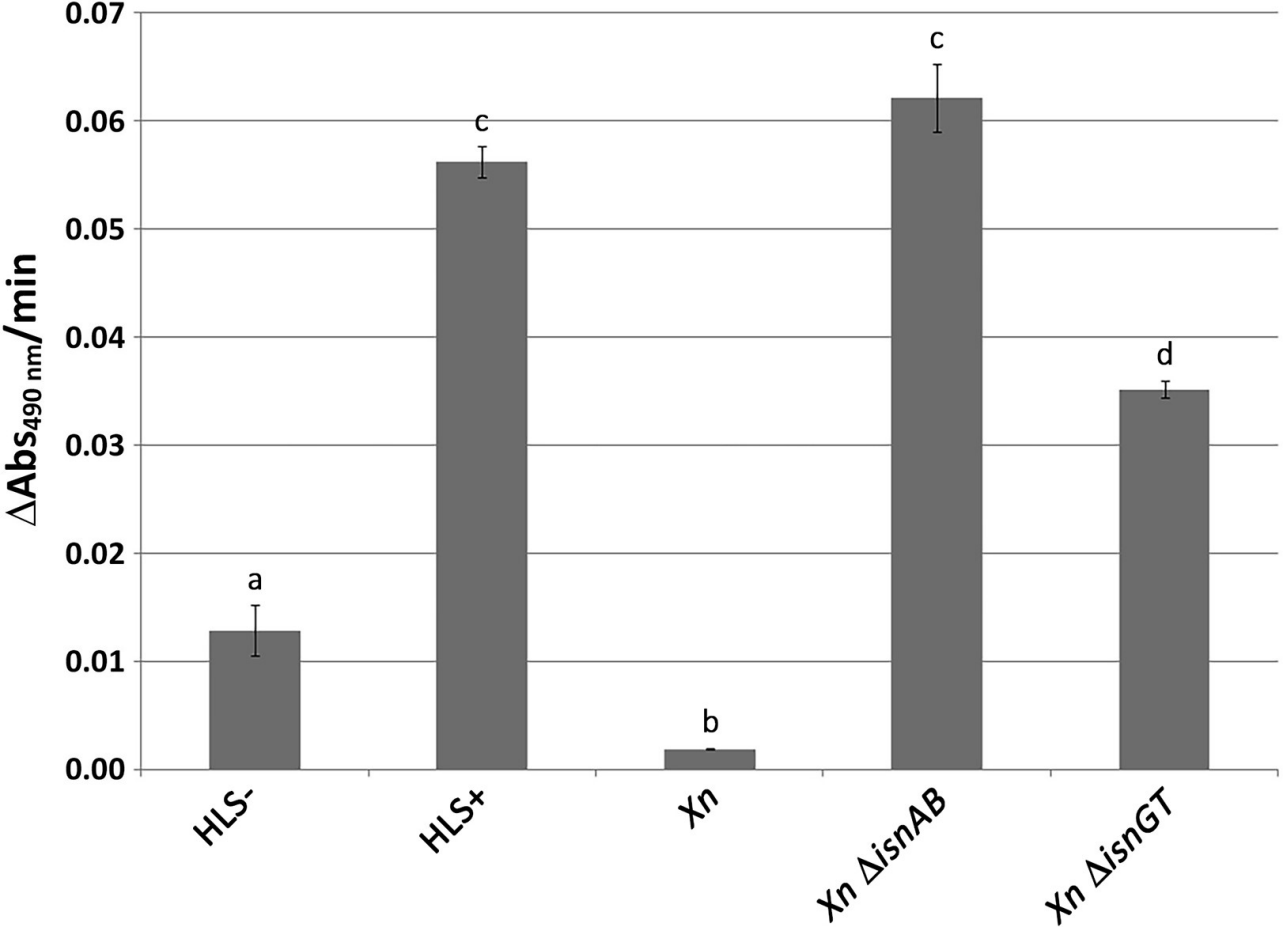
*S*. *frugiperda* phenoloxidase inhibition by culture supernatants of the *X*. *nematophila* ATCC19061^T^ wild-type strain, and those of the Δ*isnAB* and Δ*GT* mutants. Phenoloxidase specific activity is expressed as the change in absorbance as a function of time in the presence of the *S*. *frugiperda* hemocyte lysate supernatant (ΔA490/min). The HLS- sample was not activated with α-chymotrypsin and the HLS+ sample was placed in contact with sterile LB broth rather than bacterial supernatant. Mean results for three independent experiments are shown as histograms, with the standard deviations indicated. Significant differences (P<0.05) between the strains are indicated by different letters above the bars (details of data points and statistic analysis are given in S1 File).

The inhibitory effect of *Xn* cells on mushroom tyrosinase is dependent on the *Xn* rhabduscin gene cluster [27]. We evaluated the inhibitory effects of the bacterium on the phenoloxidase of a major crop pest, by developing an inhibition assay using hemocyte lysate supernatant (HLS) from *Sf*. In this assay, we measured phenoloxidase activity after incubation with bacterial culture supernatant. Unactivated *Sf* HLS (HLS-) and α-chymotrypsin-activated *Sf* HLS without bacterial culture supernatant (HLS+) were used as the negative and positive controls for phenoloxidase activity, respectively. The phenoloxidase activity observed with *Xn* culture supernatant was 6.6 fold lower than that of the HLS- control and 29 fold lower than that of the HLS+ control (Fig 2). With the Δ*isnAB* mutant, *Sf* phenoloxidase activity was similar to that of the HLS+ control, confirming the need for the *isnAB* genes for phenoloxidase inhibition. With the Δ*GT* mutant, we observed intermediate levels of *Sf* phenoloxidase activity, 1.6 fold lower than those of the HLS+ control. Thus, the products of the *isnAB* and glycosyltransferase genes are involved in the inhibition of phenoloxidase activity in *Sf*.

### The *X*. *nematophila* rhabduscin gene cluster and the inhibition of *Sf* phenoloxidase by Enterobacteriaceae species that do not inhibit phenoloxidase

**Fig 3:**
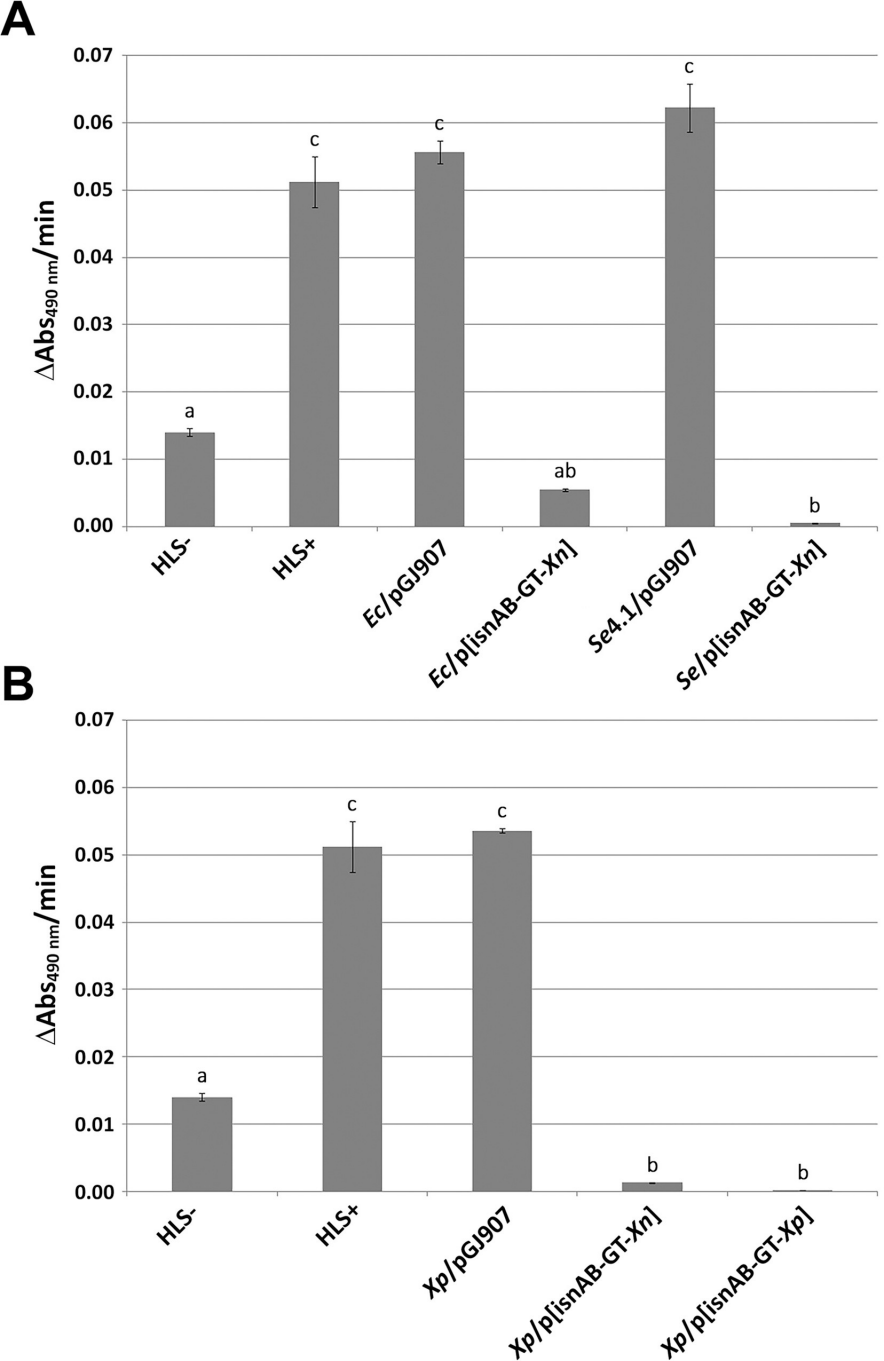
*frugiperda* phenoloxidase inhibition by the culture supernatants of several Enterobacteriaceae unable to inhibit phenoloxidase but expressing a rhabduscin gene cluster. ***S*.** Phenoloxidase specific activity was measured as described in Fig 2. (A) Assays on *Escherichia coli* XL1Blue (*Ec*/pGJ907, *E*. *coli* carrying the vector pGJ907; *Ec*/p(*isn*AB-GT-Xn), *Ec* carrying the *Xn* rhabduscin gene cluster) and *Serratia entomophila* Mor4.1. (*Se*/ pGJ907, *Se* Mor4.1 carrying the vector pGJ907; *Se*/p(*isn*AB-GT-Xn), *Se* carrying the *Xn* rhabduscin gene cluster). (B) Assays on *Xenorhabdus poinarii* G6 (*Xp*/ pGJ907, *Xp* carrying the vector pGJ907; *Xp*/p(*isn*AB-GT-Xn), *Xp* carrying the *Xn* rhabduscin gene cluster; Xp/p(*isn*AB-GT-Xp) *Xp* carrying the *Xp* rhabduscin gene cluster). Significant differences (P<0.05) between the strains are indicated by different letters above the bars (details of data points and statistic analysis are given in S2 and S3 Files).

We investigated whether the expression of the *Xn* rhabduscin gene cluster by Enterobacteriaceae species not capable of *Sf* phenoloxidase inhibition was sufficient to confer this ability. We first assessed *Sf* phenoloxidase inhibition with culture supernatants of the cloning strain *E*. *coli* XL1-Blue (*Ec*), the entomopathogenic strain *Serratia entomophila* MOR4.1 (*Se*) [33] and the attenuated virulent strain *Xenorhabdus poinarii* G6 (*Xp*) [38], each harboring the empty medium-copy number plasmid pGJ907. By contrast with what we previously observed with *Xn*, when the supernatants of *Ec*, *Se* and *Xp* harbouring the empty plasmid were used, a significant PO activity was observed similar to that observed in the positive control HLS+ (Ec/pGJ907, Se4.1/pGJ907, Xp/pGJ907 in Fig 3A and 3B). *Ec*, *Se* and *Xp* were therefore unable to inhibit *Sf* phenoloxidase activity.

We then placed the *Xn isnAB-GT* cluster under the control of the inducible P*tet* promoter of the pGJ907 plasmid. We transferred the resulting construct into *Ec*, *Se* and *Xp*. The three recombinant strains obtained, *Ec*/p[*isnAB*-*GT*-*Xn*], *Se*/p[*isnAB*-*GT*-*Xn*] and *Xp*/p[*isnAB*-*GT*-*Xn*], were able to inhibit phenoloxidase activity (Fig 3A and 3B). Heterologous expression of the *Xn* rhabduscin gene cluster is, therefore, sufficient to confer an ability to inhibit the *Sf* phenoloxidase by the culture supernatants of Enterobacteriaceae species that do not usually inhibit this enzyme.

#### The structural genes of the *X*. *poinarii* rhabduscin gene cluster are functional

**Fig 4:**
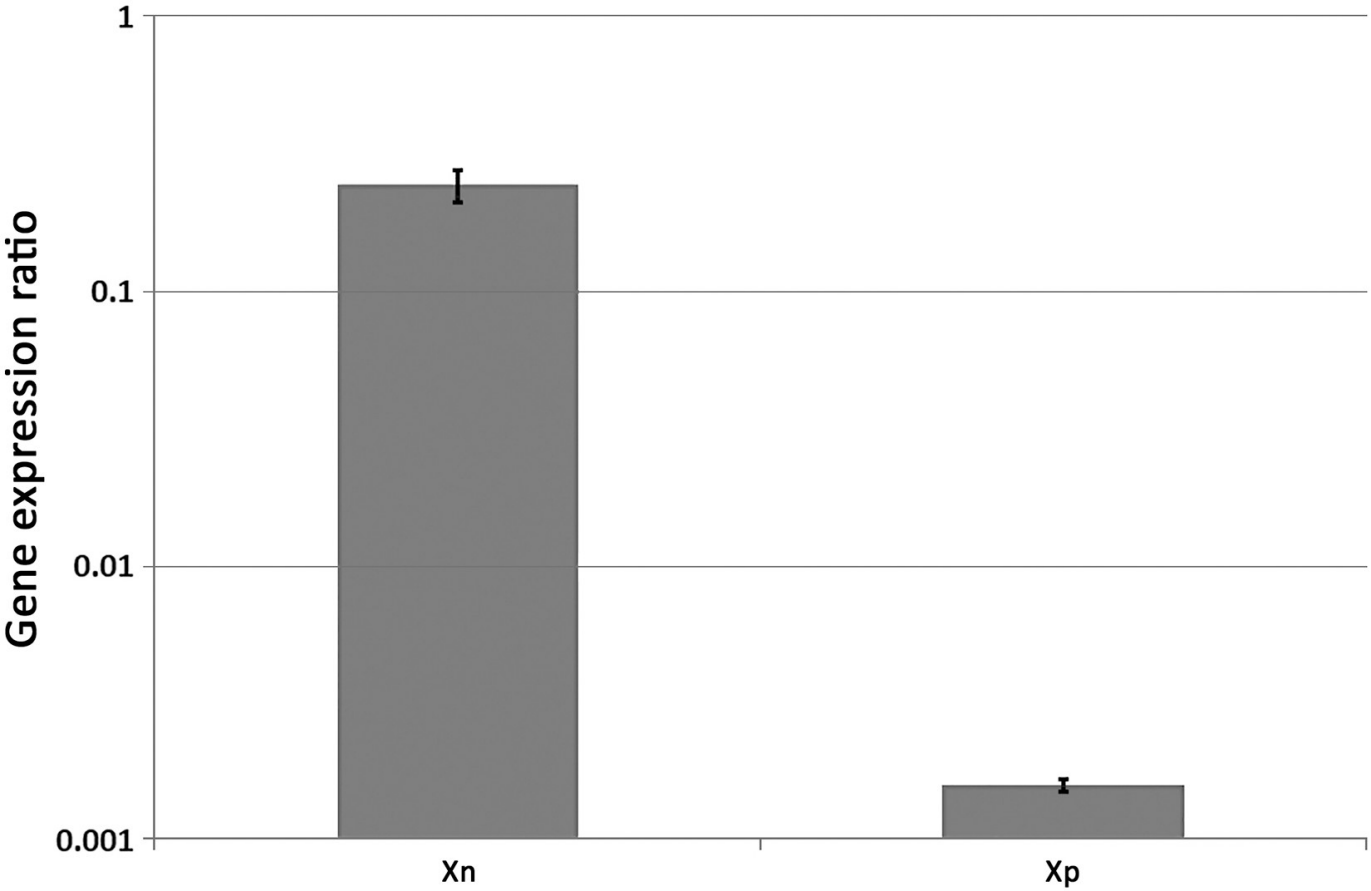
Transcription of the *isnA* gene in exponential growth-phase of *X*. *nematophila* ATCC19061 and *X*. *poinarii* G6 LB broth cultures. Total RNA from exponentially growing cultures of *X*. *nematophila* ATCC19061 (*Xn*) and *X*. *poinarii* G6 (*Xp*) strains were used for RT-qPCR analysis with internal primers specific for the *isnA* gene. Data are presented as a ratio of values for the target gene/values for the reference gene (*recA*). Relative quantification was performed in triplicate with LightCycler 480 software. The bars indicate the standard errors calculated with this software.

*Xp* culture supernatants did not inhibit *Sf* phenoloxydase activity, but we identified an ortholog of the *Xn* rhabduscin gene cluster in the *Xp* genome [38]. The *isnA*, *isnB* and *GT* orthologs had the same gene order as in the *Xn* genome [44] and the deduced amino-acid sequences of their products were 84%, 74% and 76% identical to those of the IsnA, IsnB and GT proteins of *Xn*, respectively. We compared the levels of *isnA* gene transcription in exponentially growing cultures of *Xn* and *Xp* cells in LB broth by performing reverse-transcription followed by real-time PCR. Transcription levels for the *isnA* gene were 158 times higher in *Xn* than in *Xp* (Fig 4), suggesting a transcription defect for the *Xp isnAB-GT* cluster. We introduced the *Xp isnAB-GT* cluster under the control of the inducible P*tet* promoter into the pGJ907 plasmid and transferred the resulting construct into *Xp*. *Sf* phenoloxidase activity was inhibited similarly by culture supernatants of *Xp*/p[*isnAB*-*GT*-*Xp*] and *Xp*/p[*isnAB*-*GT*-*Xn*] (Fig 3B). Thus, the endogenous promoter of the *Xp* rhabduscin gene cluster is not active, but the structural genes of the *Xp* rhabduscin gene cluster are functional and sufficient to inhibit *Sf* phenoloxidase activity.

### Pseudogenization of the *X*. *poinarii* rhabduscin gene cluster by insertion of a short element into the promoter region

**Fig 5:**
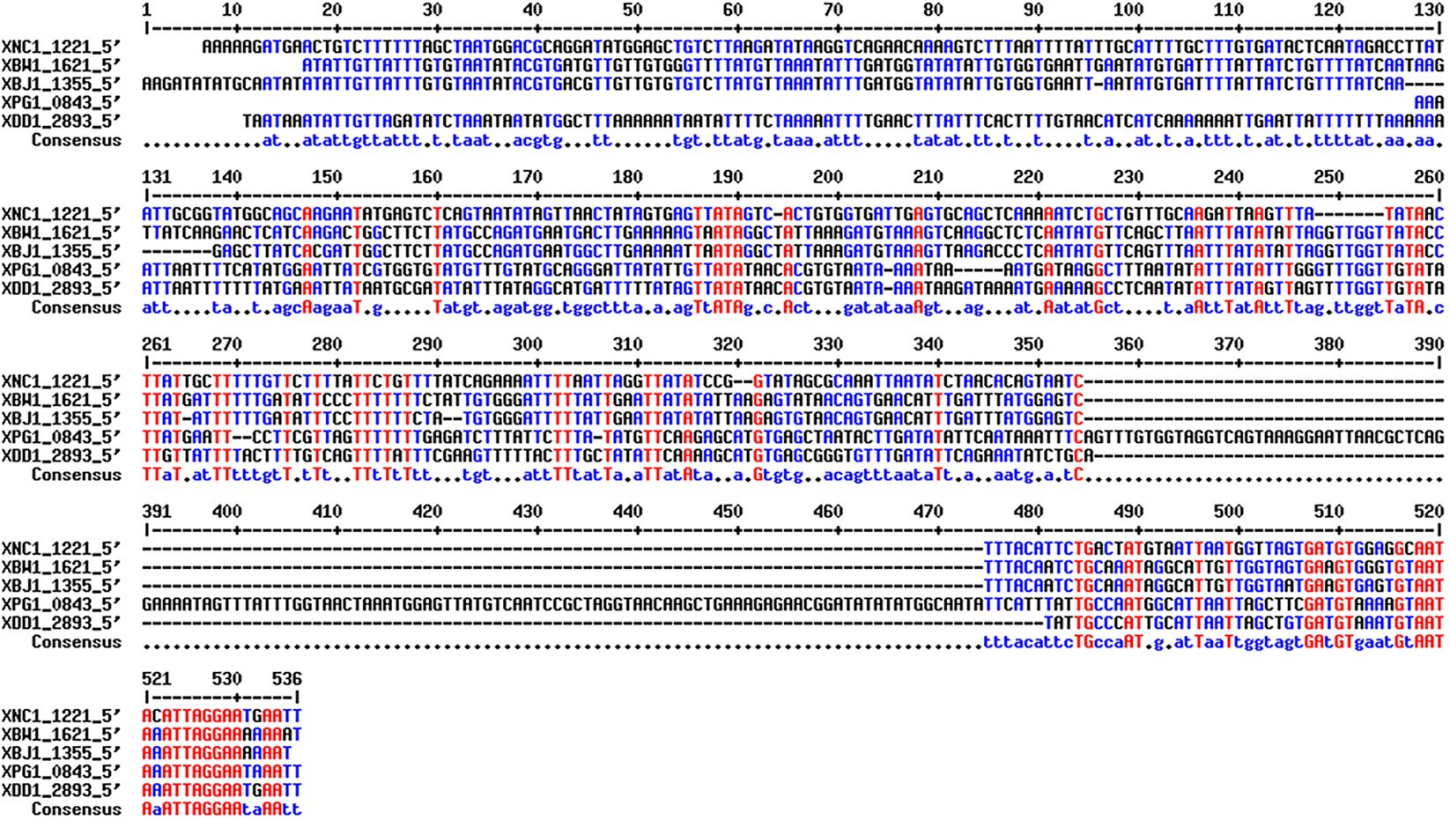
Pseudogenization of the promoter region of the *Xp* rhabduscin gene cluster by insertion of a 120-nucleotide element. Alignment of the 400 nucleotides upstream from the predicted ATG codon of five *isnA* orthologs: XNC1_1221 (*X*. *nematophila* ATCC19061^T^), XPG1_0843 (*X*. *poinarii* G6), XBW1_1621 (*X*. *bovienii* CS03), XBJ1_1355 (*X*. *bovienii* SS-2004), and XDD1_2893 (*X*. *doucetiae* FRM16). Nucleotides conserved in five, four and fewer than four genomes are shown in red, blue and black, respectively. The consensus sequence is shown below the alignment. A 120-nucleotide element interrupts the promoter region of *isnA* in the *X*. *poinarii* G6 genome.

We investigated the inactivation of the promoter of the *Xp* rhabduscin gene cluster, by generating nucleotide sequence alignments for the 400 nucleotides upstream from the predicted ATG codon of *isnA* from the genomes of *Xp*, *Xn* and three other *Xenorhabdus* strains for which whole-genome sequences were available: *X*. *bovienii* CS03, *X*. *bovienii* SS-2004, and *X*. *doucetiae* FRM16 [38,44,45]. The *Xp* strain was the only strain carrying a 120 nucleotide-long element interrupting the *isnA* promoter region (Fig 5). The sequence of this element was unique within the *Xp* genome and not reported in public databases. It displayed no remarkable features, such as repeats or palindromes.

## Discussion

We show here that the *X*. *nematophila* rhabduscin gene cluster is not necessary for bacterial pathogenicity in insects of the genus *Spodoptera*. However, this cluster is sufficient for inhibition of the *S*. *frugiperda* (*Sf*) phenoloxidase, a major component of the humoral immune system of the insect. This phenotype was confirmed in *X*. *nematophila* ATCC19061^T^ (*Xn*), but also by heterologous expression of the *Xn* rhabduscin gene cluster in species of Enterobacteriaceae devoid of any natural ability to inhibit phenoloxidase.

We developed a phenoloxidase assay for hemocyte lysate supernatants from *Sf* similar to that previously described for *Locusta migratoria* (Orthoptera:Acrididae) [37] and we used it to assess the inhibition of phenoloxidase activity by bacterial culture supernatants. As previously shown with washed bacterial cells and mushroom tyrosinase [27], the wild-type *Xn* strain inhibited phenoloxidase activity whereas the *Xn* Δ*isnAB* mutant did not. In our assay, the *Xn* Δ*GT* mutant displaying an accumulation of the aglycone intermediate of the rhabduscin biosynthesis pathway had an intermediate inhibition phenotype. By contrast, when washed bacterial cells were used, the *Xn* Δ*GT* mutant had a phenoloxidase inhibition, phenotype similar to that of the wild-type *Xn* strain [27]. Our test is therefore able to detect more subtle variations. Glycosylation is one of the most important modification processes undergone by small molecules, and it increases the solubility, stability, and bioactivity of the parent molecule [46]. Rhabduscin has been shown to be located on both the bacterial surface and in cell-free culture supernatants [27]. Glycosyltransferase activity may be required to stabilize rhabduscin when it is not associated with the cell envelope.

Two of the Enterobacteriaceae species devoid of a natural ability to inhibit phenoloxidase tested here are entomopathogenic bacteria: *Se* and *Xp*. *Serratia entomophila* species was initially described as a specialist pathogen of the coleopteran insect pest, *Costelytra zealandica*, which causes “amber disease” in the grasslands of New Zealand [47]. The strain used here, *S*. *entomophila* MOR4.1 (*Se*), was recently isolated from the larva of another Coleoptera, *Phyllophaga blanchardi*, from a Mexican maize field [33]. *Se* is considered to be a Coleoptera-specific pathogen; it resides in the larval gut for several weeks, inhibiting feeding activity, and it then enters the hemocoel and kills the larva [33]. This late interaction with the hemolymph probably explains the lack of phenoloxidase-inhibiting factors in this bacterium.

By contrast, most species of *Xenorhabdus* can block phenoloxidase activity [24,27,37]. This probably enables them to avoid the effects of prophenoloxidase system activation when the symbiotic nematodes located in the digestive tract of insect larvae inject the bacteria from their gut into the insect hemolymph [16,17]. The only exception reported to date in this genus is *Xenorhabdus innexi*, which cannot block phenoloxidase activity and lacks the entire rhabduscin gene cluster [48]. *Xp* is different because it harbors complete and functional *isnA*, *isnB* and *GT* coding sequences, but there is a 120-bp element inserted into the promoter region of *isnA*, impairing transcription of the rhabduscin gene cluster. This finding is consistent with recent pseudogenization of the *Xp* rhabduscin gene cluster. The genome of *Xp* is markedly small, and *Xp* genome reduction has previously been shown to occur following the excision of genomic blocks from the flexible genome [38]. In bacteria, pseudogenes are rapidly removed by deletion [49,50]. The insertion of this 120 bp element may be the first step towards excision of the rhabduscin gene cluster. Genomic reduction may, therefore, still be ongoing in *Xp*. This evolutionary pathway probably reflects greater reliance on the nematode and/or insect hosts than in other *Xenorhabdus* species. For example, the surface coat proteins of the symbiotic nematode carrying *X*. *poinarii* have phenoloxidase-inhibiting activity [51].

We were unable to reproduce previous data obtained with waxmoth larvae [27]. It is difficult to standardize *G*. *mellonella* pathoassays because it is hard to establish the instar of the larvae, and more repetitions are required than that performed by Crawford and coworkers. In the lepidopteran pests *S*. *littoralis* (*Sl*) and *Sf*, rhabduscin synthesis mutants are at least as pathogenic as the wild-type strain. The pathology assay for *Sl* has been standardized for some time in our laboratory (see for example [16,37,43]). *Sl* and *Sf* are also considered much more resistant to *Xenorhabdus* infections than *G*. *mellonella* [38,43]. Moreover, melanization products and by-products are highly toxic to insect larvae [4,6]. The strong immune response triggered by *Xn* rhabduscin mutants in *Sl* and *Sf* is therefore probably more toxic than that triggered by the wild-type strain. This result highlights the importance of the regulation of phenoloxidase synthesis. In situations other than infection, the prophenoloxidase activation cascade and active phenoloxidase are subject to tight temporal and spatial control mediated by endogenous serine proteinases and specific phenoloxidase inhibitors, respectively [4].

The role of rhabduscin of *X*. *nematophila* virulence in insects remains unclear. Two hypotheses could explain the lack of virulence difference between wild type and mutants of rhabduscin synthesis cluster. First, this strain displays a potential for multifactorial virulence, as suggested by exploration of the *X*. *nematophila* genome [44]. These multiple and sometimes redundant virulence factors likely confer to *X*. *nematophila* a panoply of virulence strategies that at the end lead to the same result, the death of the insect. In the phylogenetically close entomopathogenic bacterium, *Photorhabdus luminescens*, a type three secretion system mutant is altered in nodule formation capacity, but the mutant is not affected in the whole virulence [52]. It has also been reported that microbial secondary metabolites as isocyanides have a broad repertoire of biological properties including cytotoxicity and antibacterial ability [53]. Since rhabduscin has an isocyanide moiety, another hypothesis is possible that rhabduscin fulfills other roles in the whole life-cycle process of the nematode-bacteria pair as the control of microbial populations inside the insect cadaver.

In conclusion, infection is the result of a multifactorial process dependent on both pathogen virulence and host susceptibility. Researchers are becoming increasingly mindful of the differences between entomopathogenic strains [37,38,43,45] and insect hosts [54] in studies of pathogenic interactions between insects and bacteria. Assays should therefore be developed on non-model hosts, such as the new invasive African *S*. *frugiperda* species in this study.

## Supporting information

S1 File Data points and statistic analysis for Fig 2. (PDF)

S2 File Data points and statistic analysis for Fig 3A. (PDF)

S3 File Data points and statistic analysis for Fig 3B. (PDF)
