# Supplementary material for: Inhibition of Spodoptera frugiperda phenoloxidase activity by the products of the Xenorhabdus rhabduscin gene cluster
Source: PLoS One. 2019 Feb 22;14(2):e0212809. doi: 10.1371/journal.pone.0212809 (PMC6386379; doi:10.1371/journal.pone.0212809)
Supplement: S1 File — (PDF) [file pone.0212809.s001.pdf]

Figure 2.

Data from three experiments

OD5-OD1/9min *1					
Col. title	**HLS-**	**HLS+**	***Xn***	***Xn ΔisnAB***	***Xn ΔisnGT***
**1**	0.01207	0.0533	0.0018	0.0596	0.0334
**2**	0.0086	0.059	0.002	0.0693	0.0366
**3**	0.018	0.0564	0.002	0.0575	0.0355
**Mean**	0.01289	0.05623333	0.00193333	0.06213333	0.03516667
**Standard deviation (SD)**	0.004753	0.002854	0.0001155	0.006295	0.001626
**Sample size (N)**	3	3	3	3	3

*1 The optical density of the sample at time 5 minus the optical density of the sample at time 1 divided by 9 minutes. The average of the sample represents the average of three experiments, where each experiment represents in the same way the average of two readings of each sample.

The differences among samples were assessed by the Tukey-Kramer Multiple Comparisons Test. The null hypothesis (Ho) for this test states that there are no differences among the means of the compared samples. The null hypothesis was contrasted against the alternative hypothesis (Ha) indicating that there are differences among the means of the samples evaluated. The GraphPad InStat3 software was used to make statistical tests.

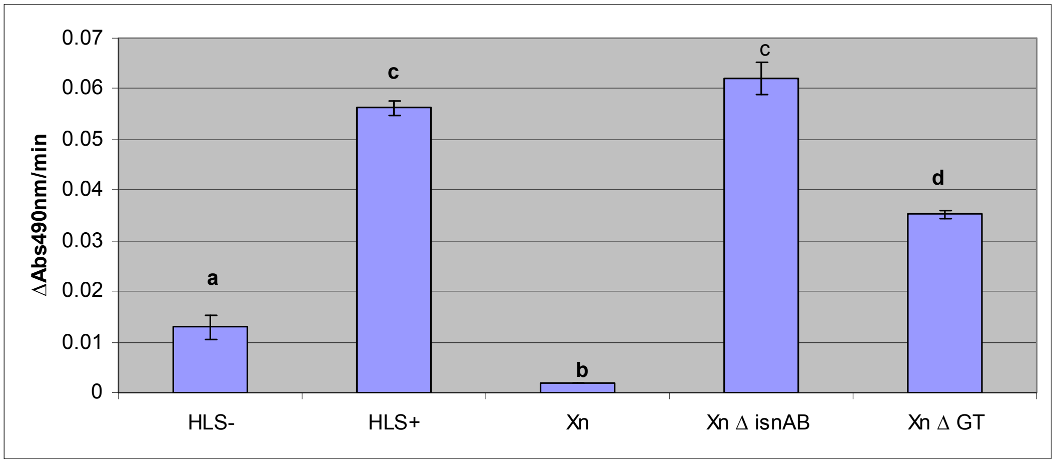

Significant difference among the mean of the samples is indicated by different letter above the bars.

One-way Analysis of Variance (ANOVA)

The P value is < 0.0001, considered extremely significant.
Variation among column means is significantly greater than expected by chance.

Tukey-Kramer Multiple Comparisons Test

If the value of q is greater than 4.654 then the P value is less than 0.05.

Comparison	Mean Difference	q	P value
HLS- vs HLS+	-0.04334	19.645	*** P<0.001
HLS- vs Xn	0.01096	4.966	* P<0.05
HLS- vs Xn ΔisnAB	-0.04924	22.319	*** P<0.001
HLS- vs Xn ΔisnGT	-0.02228	10.097	*** P<0.001
HLS+ vs Xn	0.05430	24.611	*** P<0.001
HLS+ vs Xn ΔisnAB	-0.00590	2.674	ns P>0.05
HLS+ vs Xn ΔisnGT	0.02107	9.548	*** P<0.001
Xn vs Xn ΔisnAB	-0.06020	27.285	*** P<0.001
Xn vs Xn ΔisnGT	-0.03323	15.063	*** P<0.001
Xn ΔisnAB vs Xn ΔisnGT	0.02697	12.222	*** P<0.001

Difference	Mean Difference	95% Confidence Interval From	To
HLS- - HLS+	-0.04334	-0.05361	-0.03308
HLS- - Xn	0.01096	0.0006885	0.02122
HLS- - Xn ΔisnAB	-0.04924	-0.05951	-0.03898
HLS- - Xn ΔisnGT	-0.02228	-0.03254	-0.01201
HLS+ - Xn	0.05430	0.04403	0.06457
HLS+ - Xn ΔisnAB	-0.005900	-0.01617	0.004368
HLS+ - Xn ΔisnGT	0.02107	0.01080	0.03133
Xn - Xn ΔisnAB	-0.06020	-0.07047	-0.04993
Xn - Xn ΔisnGT	-0.03323	-0.04350	-0.02297
Xn ΔisnAB - Xn ΔisnGT	0.02697	0.01670	0.03723
