# Supplementary material for: Inhibition of Spodoptera frugiperda phenoloxidase activity by the products of the Xenorhabdus rhabduscin gene cluster
Source: PLoS One. 2019 Feb 22;14(2):e0212809. doi: 10.1371/journal.pone.0212809 (PMC6386379; doi:10.1371/journal.pone.0212809)
Supplement: S2 File — (PDF) [file pone.0212809.s002.pdf]

Figure 3A

Data from three experiments

Col Title	HLS-	HLS+	Ec/pGJ907	Ec/p[isnAB-GT-Xn]	Se4.1/pGJ907	Se/p[isnAB-GT-Xn]
	**OD5-OD1/9min *1**					
**1**	0.0151	0.0449	0.0532	0.005	0.0545	0.0005
**2**	0.0129	0.049	0.0543	0.0057	0.0636	0.0006
**3**	0.0141	0.0597	0.0594	0.0057	0.0685	0.0005
**Mean**	0.01403333	0.0512	0.05563333	0.00546667	0.0622	0.00053333
**Standard deviation (SD)**	0.001102	0.007641	0.003308	0.0004041	0.007104	5.77E-05
**Sample size (N)**	3	3	3	3	3	3

*1 The optical density of the sample at time 5 minus the optical density of the sample at time 1 divided by 9 minutes. The average of the sample represents the average of three experiments, where each experiment represents in the same way the average of two readings of each sample.

The differences among samples were assessed by the Tukey-Kramer Multiple Comparisons Test. The null hypothesis (Ho) for this test states that there are no differences among the means of the compared samples. The null hypothesis was contrasted against the alternative hypothesis (Ha) indicating that there are differences among the means of the samples evaluated. The GraphPad InStat3 software was used to make statistical tests.

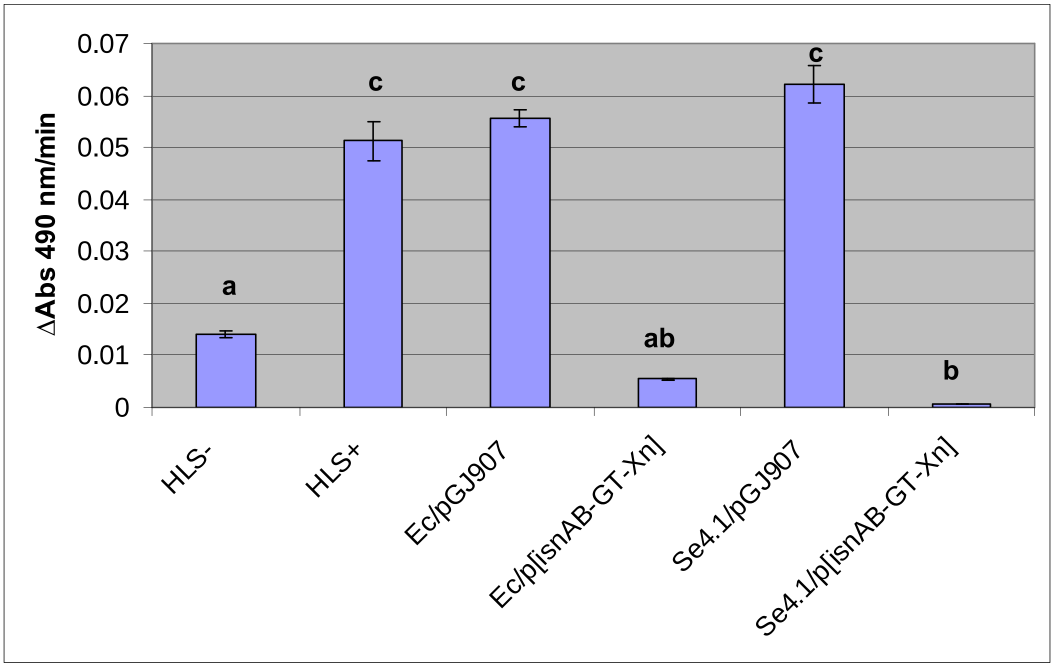

Significant difference among the mean of the samples is indicated by different letter above the bars.

One-way Analysis of Variance (ANOVA)

The P value is < 0.0001, considered extremely significant.
Variation among column means is significantly greater than expected by chance.

Tukey-Kramer Multiple Comparisons Test

If the value of q is greater than 4.751 then the P value is less than 0.05.

Comparison	Mean Difference	q	P value
HLS- vs HLS+	-0.03717	14.324	*** P<0.001
HLS- vs Ec/pGJ907	-0.04160	16.033	*** P<0.001
HLS- vs Ec/p[isnAB-GT-Xn]	0.008567	3.302	ns P>0.05
HLS- vs Se4.1/pGJ907	-0.04817	18.564	*** P<0.001
HLS- vs Se/p[isnAB-GT-Xn]	0.01350	5.203	* P<0.05
HLS+ vs Ec/pGJ907	-0.004433	1.709	ns P>0.05
HLS+ vs Ec/p[isnAB-GT-Xn]	0.04573	17.626	*** P<0.001
HLS+ vs Se4.1/pGJ907	-0.01100	4.239	ns P>0.05
HLS+ vs Se/p[isnAB-GT-Xn]	0.05067	19.527	*** P<0.001
Ec/pGJ907 vs Ec/p[isnAB-GT-Xn]	0.05017	19.334	*** P<0.001
Ec/pGJ907 vs Se4.1/pGJ907	-0.006567	2.531	ns P>0.05
Ec/pGJ907 vs Se/p[isnAB-GT-Xn]	0.05510	21.236	*** P<0.001
Ec/p[isnAB-GT-Xn] vs Se4.1/pGJ907	-0.05673	21.865	*** P<0.001
Ec/p[isnAB-GT-Xn] vs Se/p[isnAB-GT-Xn]	0.004933	1.901	ns P>0.05
Se4.1/pGJ907 vs Se/p[isnAB-GT-Xn]	0.06167	23.767	*** P<0.001

Difference	Mean Difference	95% Confidence Interval From	To
HLS- - HLS+	-0.03717	-0.04949	-0.02484
HLS- - Ec/pGJ907	-0.04160	-0.05393	-0.02927
HLS- - Ec/p[isnAB-GT-Xn]	0.008567	-0.003761	0.02089
HLS- - Se4.1/pGJ907	-0.04817	-0.06049	-0.03584
HLS- - Se/p[isnAB-GT-Xn]	0.01350	0.001173	0.02583
HLS+ - Ec/pGJ907	-0.004433	-0.01676	0.007894
HLS+ - Ec/p[isnAB-GT-Xn]	0.04573	0.03341	0.05806
HLS+ - Se4.1/pGJ907	-0.01100	-0.02333	0.001327
HLS+ - Se/p[isnAB-GT-Xn]	0.05067	0.03834	0.06299
Ec/pGJ907 - Ec/p[isnAB-GT-Xn]	0.05017	0.03784	0.06249
Ec/pGJ907 - Se4.1/pGJ907	-0.006567	-0.01889	0.005761
Ec/pGJ907 - Se/p[isnAB-GT-Xn]	0.05510	0.04277	0.06743
Ec/p[isnAB-GT-Xn] - Se4.1/pGJ907	-0.05673	-0.06906	-0.04441
Ec/p[isnAB-GT-Xn] - Se/p[isnAB-GT-Xn]	0.004933	-0.007394	0.01726
Se4.1/pGJ907 - Se/p[isnAB-GT-Xn]	0.06167	0.04934	0.07399
