# Supplementary material for: Inhibition of Spodoptera frugiperda phenoloxidase activity by the products of the Xenorhabdus rhabduscin gene cluster
Source: PLoS One. 2019 Feb 22;14(2):e0212809. doi: 10.1371/journal.pone.0212809 (PMC6386379; doi:10.1371/journal.pone.0212809)
Supplement: S3 File — (PDF) [file pone.0212809.s003.pdf]

Fig. 3B

Data from three experiments

	OD5-OD1/9min *1				
**Col. Title**	**HLS-**	**HLS+**	**Xp/pGJ907**	**Xp/p[isnAB-GT-Xn]**	**Xp/p[isnAB-GT-Xp]**
**1**	0.0151	0.0449	0.0461	0.0011	0.0002
**2**	0.0129	0.049	0.0551	0.0014	0.0002
**3**	0.0141	0.0597	0.0595	0.0014	0.0002
**Mean**	0.01403333	0.0512	0.05356667	0.0013	0.0002
**Standard deviation (SD)**	0.001102	0.007641	0.00683	0.0001732	0
**Sample size (N)**	3	3	3	3	3

*1 The optical density of the sample at time 5 minus the optical density of the sample at time 1 divided by 9 minutes. The average of the sample represents the average of three experiments, where each experiment represents in the same way the average of two readings of each sample.

The differences among samples were assessed by the Tukey-Kramer Multiple Comparisons Test. The null hypothesis (Ho) for this test states that there are no differences among the means of the compared samples. The null hypothesis was contrasted against the alternative hypothesis (Ha) indicating that there are differences among the means of the samples evaluated. The GraphPad InStat3 software was used to make statistical tests.

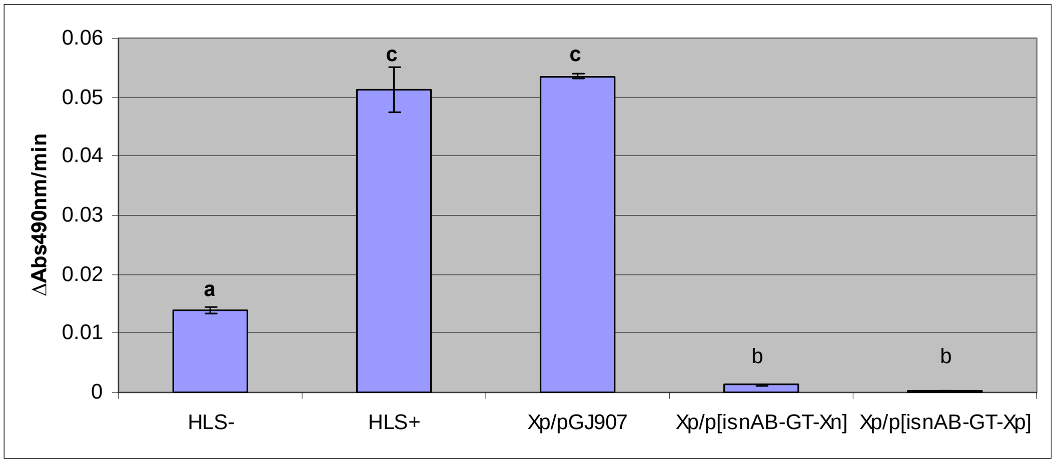

Significant difference among the mean of the samples is indicated by different letter above the bars.

One-way Analysis of Variance (ANOVA)

The P value is < 0.0001, considered extremely significant.
Variation among column means is significantly greater than expected by chance.

Tukey-Kramer Multiple Comparisons Test

If the value of q is greater than 4.654 then the P value is less than 0.05.

Comparison	Mean Difference	q	P value
HLS- vs HLS+	-0.03717	13.962	*** P<0.001
HLS- vs Xp/pGJ907	-0.03953	14.851	*** P<0.001
HLS- vs Xp/p[isnAB-GT-Xn]	0.01273	4.784	* P<0.05
HLS- vs Xp/p[isnAB-GT-Xp]	0.01383	5.197	* P<0.05
HLS+ vs Xp/pGJ907	-0.002367	0.8891	ns P>0.05
HLS+ vs Xp/p[isnAB-GT-Xn]	0.04990	18.746	*** P<0.001
HLS+ vs Xp/p[isnAB-GT-Xp]	0.05100	19.159	*** P<0.001
Xp/pGJ907 vs Xp/p[isnAB-GT-Xn]	0.05227	19.635	*** P<0.001
Xp/pGJ907 vs Xp/p[isnAB-GT-Xp]	0.05337	20.048	*** P<0.001
Xp/p[isnAB-GT-Xn] vs Xp/p[isnAB-GT-Xp]	0.001100	0.4132	ns P>0.05

Difference	Mean Difference	95% Confidence Interval From	To
HLS- - HLS+	-0.03717	-0.04956	-0.02478
HLS- - Xp/pGJ907	-0.03953	-0.05192	-0.02714
HLS- - Xp/p[isnAB-GT-Xn]	0.01273	0.0003448	0.02512
HLS- - Xp/p[isnAB-GT-Xp]	0.01383	0.001445	0.02622
HLS+ - Xp/pGJ907	-0.002367	-0.01476	0.01002
HLS+ - Xp/p[isnAB-GT-Xn]	0.04990	0.03751	0.06229
HLS+ - Xp/p[isnAB-GT-Xp]	0.05100	0.03861	0.06339
Xp/pGJ907 - Xp/p[isnAB-GT-Xn]	0.05227	0.03988	0.06466
Xp/pGJ907 - Xp/p[isnAB-GT-Xp]	0.05337	0.04098	0.06576
Xp/p[isnAB-GT-Xn] - Xp/p[isnAB-GT-Xp]	0.001100	-0.01129	0.01349
